# Human Prolactin Point Mutations and Their Projected Effect on Vasoinhibin Generation and Vasoinhibin-Related Diseases

**DOI:** 10.3389/fendo.2017.00294

**Published:** 2017-11-06

**Authors:** Jakob Triebel, Christin J. Friedrich, Andreas Leuchs, Gonzalo Martínez de la Escalera, Carmen Clapp, Thomas Bertsch

**Affiliations:** ^1^Institute for Clinical Chemistry, Laboratory Medicine and Transfusion Medicine, Nuremberg General Hospital, Paracelsus Medical University, Nuremberg, Germany; ^2^Instituto de Neurobiología, Universidad Nacional Autónoma de México (UNAM), Querétaro, Mexico

**Keywords:** prolactin mutations, vasoinhibins, prolactin/vasoinhibin axis, peripartum cardiomyopathy, 16 kDa prolactin

## Abstract

**Background:**

A dysregulation of the generation of vasoinhibin hormones by proteolytic cleavage of prolactin (PRL) has been brought into context with diabetic retinopathy, retinopathy of prematurity, preeclampsia, pregnancy-induced hypertension, and peripartum cardiomyopathy. Factors governing vasoinhibin generation are incompletely characterized, and the composition of vasoinhibin isoforms in human tissues or compartments, such as the circulation, is unknown. The aim of this study was to determine the possible contribution of PRL point mutations to the generation of vasoinhibins as well as to project their role in vasoinhibin-related diseases.

**Methods:**

Prolactin sequences, point mutations, and substrate specificity information about the PRL cleaving enzymes cathepsin D, matrix metalloproteinases 8 and 13, and bone-morphogenetic protein 1 were retrieved from public databases. The consequences of point mutations in regard to their possible effect on vasoinhibin levels were projected on the basis of a score indicating the suitability of a particular sequence for enzymatic cleavage that result in vasoinhibin generation. The relative abundance and type of vasoinhibin isoforms were estimated by comparing the relative cleavage efficiency of vasoinhibin-generating enzymes.

**Results:**

Six point mutations leading to amino acid substitutions in vasoinhibin-generating cleavage sites were found and projected to either facilitate or inhibit vasoinhibin generation. Four mutations affecting vasoinhibin generation in cancer tissues were found. The most likely composition of the relative abundance of vasoinhibin isoforms is projected to be 15 > 17.2 > 16.8 > 17.7 > 18 kDa vasoinhibin.

**Conclusion:**

Prolactin point mutations are likely to influence vasoinhibin levels by affecting the proteolysis efficiency of vasoinhibin-generating enzymes and should be monitored in patients with vasoinhibin-related diseases. Attempts to characterize vasoinhibin-related diseases should include the 15, 17.2, 16.8, 17.7, and 18 kDa vasoinhibin isoforms.

## Introduction

The regulation of blood vessel growth, pressure, permeability, and dilation is essential for all vertebrate species and is in part executed by hormones such as vasoinhibins (1–3). Apart from their vascular effects, vasoinhibins exert non-vascular actions, which include the stimulation of vasopressin release (4), proinflammatory actions (5) thrombolytic effects (6), and the stimulation of anxiety- and depression-related behaviors (7). Vasoinhibins are generated by the proteolytic cleavage of their precursor molecule, the pituitary hormone prolactin (PRL). This cleavage takes place in various anatomical compartments and tissues, such as the pituitary gland (8), the retina (9), the heart (10), and the cartilage (11). As the cleaving enzymes, matrix metalloproteinases (MMP), cathepsin D, and bone-morphogenetic protein 1 (BMP-1) utilize multiple cleavage sites along the PRL amino acid sequence, the molecular mass of the vasoinhibin isoforms varies from 11 to 18 kDa (11–13). The regulation of vasoinhibins occurs at the hypothalamic, pituitary, and peripheral level demonstrating typical features of an endocrine axis. Consequently, the organizational principle of the regulation of vasoinhibins has been described as the PRL/vasoinhibin axis (14).

Dysregulation of the PRL/vasoinhibin axis has been brought into context with diabetic retinopathy, retinopathy of prematurity (9, 15, 16), preeclampsia, and pregnancy-induced hypertension (10, 17–21). In regard to the PRL/vasoinhibin axis, the shared hallmark of these diseases is the dysregulation of vasoinhibin levels, that means they may either be too low to execute a physiological function—such as the suppression of angiogenesis in the eye—or too high, which may lead to detrimental effects on the microvasculature of the heart, as proposed for the etiopathology of peripartum cardiomyopathy (PPCM). The interplay between two factors appears to be decisive for such dysregulation: the availability of the substrate, that is the level of pituitary PRL secretion, and the activity of the PRL-cleaving enzyme(s), cathepsin D, MMP, and BMP-1. Here, it is hypothesized that a third factor may be of relevance for the generation of vasoinhibins: the PRL amino acid sequence, or, more precisely, point mutations in the PRL gene which translate into amino acid substitutions within the PRL protein sequence, with the effect of enhancing or inhibiting enzymatic cleavage and, thereby, altering the levels and type of the generated vasoinhibins.

Vasoinhibins have been detected in the circulation and in other biological fluids and tissue extracts, but the techniques used for their detection are not quantitative, nor specific enough to provide unambiguous identification of their respective isoform(s). In consequence, the concentration and type of vasoinhibin isoforms are unknown (22). This is a major obstacle limiting the characterization of vasoinhibins’ emerging roles in the abovementioned diseases (23). Besides microfluidics- and mass-spectrometry-based assays (15, 24), the only established technique to measure vasoinhibins is immunoprecipitation with anti-PRL antibodies followed by SDS-PAGE and Western blotting. Quantitative immunoassays usually employ monoclonal antibodies, but the development of anti-vasoinhibin monoclonal antibodies able to discriminate between PRL and vasoinhibins has been delayed due to uncertainties about which isoform should be targeted—a problem aggravated by the observation that the type of vasoinhibin isoform appears to vary in physiological and pathological conditions. Here, we used a bioinformatic approach to project how the enzymatic cleavage-efficiency generating vasoinhibins is affected by known PRL point mutations. Furthermore, we have estimated the relative vasoinhibin isoform composition expected on the basis of the efficacy of their generation process.

## Materials and Methods

**Figure 1:**
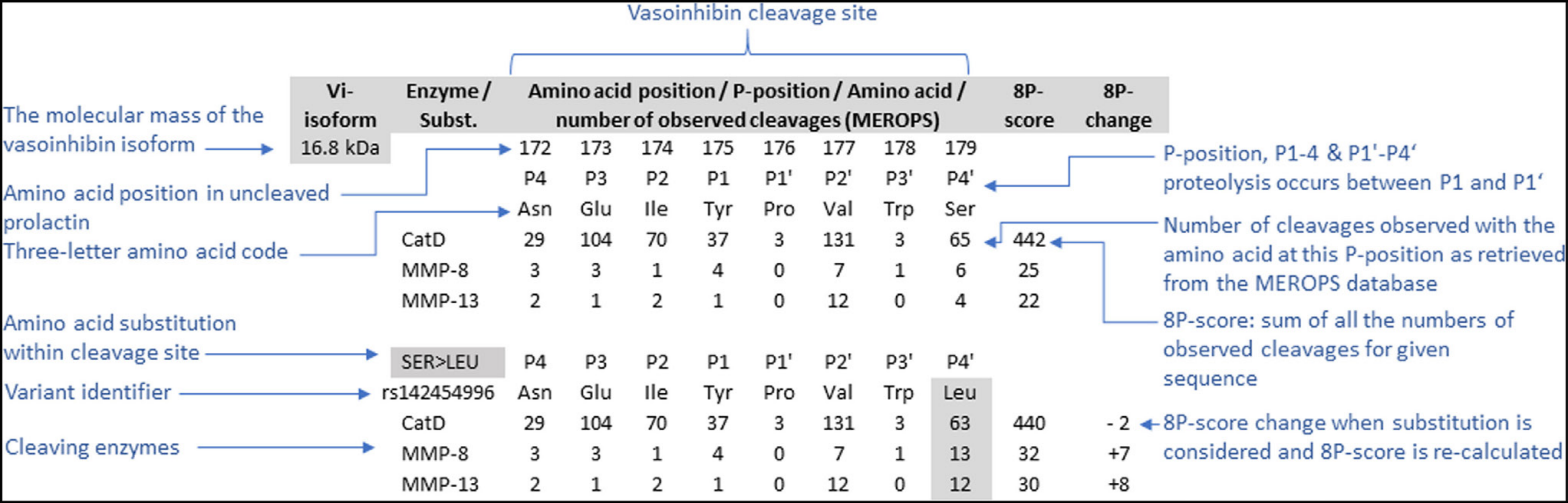
To project the consequences of single amino acid substitutions in vasoinhibin-generating cleavage sites of the prolactin amino acid sequence, an 8P-score was used. The score consists of the sum of the numbers of observed cleavages by the enzyme indicated with a particular amino acid on each of the eight positions (P1’–P4’, P1–P4), retrieved from the MEROPS database (28). The cleavage occurs between positions P1’ and P1. The effect of amino acid substitutions was projected by recalculation of the score after replacement of the amino acid and adjusting the corresponding number of observed cleavages.

Data concerning the suitability of cleavage site amino acid sequence for proteolytic cleavage by cathepsin D, MMP, and BMP-1 with subsequent generation of vasoinhibins were retrieved from databases, and a projection for single amino acid substitutions was performed. For this purpose, human PRL gene transcripts (PRL-001, transcript ID: ENST00000306482.1, PRL 202, ID: ENST00000617911.4, PRL 201, ID: ENST00000615510.4) and amino acid sequences were retrieved from ENSEMBL^1^ (25), as were missense variants, with detected point mutations leading to amino acid substitutions within the PRL sequence. The amino acid sequences of the three transcripts were aligned using MEGA, version 7 (clustal W alignment) (26). Mutations of the PRL gene in cancer were identified in ENSEMBL and details were retrieved from COSMIC (Catalogue of Somatic Mutations in Cancer^2^) (27). Furthermore, the MEROPS database of proteolytic enzymes (28), their substrates, and inhibitors^3^ was interrogated to obtain information about the cleavage site specificity of cathepsin D (MEROPS ID: A01.009), MMP-8 (M10.002), MMP-13 (M10.013) and BMP-1 (M12.005). The following five vasoinhibin isoforms, for which specific cleavage site information are available, were selected for this analysis: the 16.8, 17.2, 17.7, 18, and 15 kDa vasoinhibin isoform. Each cleavage site generating vasoinhibins was defined by the 8 amino acids neighboring the cleavage site (P1–P4, and P1’–P4’) meaning four residues toward the N-terminus and four toward the C-terminus of the uncleaved PRL sequence. The specificity matrix for each enzyme was retrieved and entries of numbers of cleavages for each amino acid at a particular position of the cleavage site were added up to build an “8P”-score—a surrogate parameter for the “suitability” of a given sequence for cleavage by the respective enzyme. Missense variants were matched with the cleavage sites of five vasoinhibin isoforms and the effect of the amino acid substitutions on the 8P-score was projected according to the MEROPS specificity matrix of the respective enzyme. An example of how the 8P-score was generated for the ancestral cleavage sites, including the effect of an amino acid substitution, is presented in Figure 1.

## Results

Six point mutations leading to a single amino acid substitution in cleavage sites affecting four vasoinhibin isoforms were found (Table 1). A SER/LEU substitution at P4’ of the cleavage site of the 16.8 kDa vasoinhibin isoform results in a slightly higher 8P-score for MMP-8 (+7 units) and MMP-13 (+8), whereas cleavage by cathepsin D is slightly reduced (−2). The TRP/CYS substitution at P3’ leads to a higher score for cathepsin D (+8), whereas the score for MMP-8 and MMP-13 demonstrates only minor and no change, respectively (+1; 0). Greater changes are observed due to the same substitution (TRP/CYS) and the SER/LEU substitution in the cleavage site of the 17.2 kDa vasoinhibin isoform. The TRP/CYS substitution leads to a reduction of the 8P-score by 21 units and the SER/LEU substitution to an increase of +69 units, both for cleavage by cathepsin D. A SER/ALA substitution in the cleavage site of the 17.7 kDa vasoinhibin isoform leads to slightly higher scores for MMP-8 (+2) and MMP-13 (+2). The MET/VAL substitution at P3 in the cleavage site for the 15 kDa vasoinhibin isoform leads to a higher score (+68) for cathepsin D. Four COSMIC point mutations causing amino acid substitutions in cleavage sites affecting the generation of three vasoinhibin isoforms were found and analyzed in the same manner (Table 2).

**Table 1:** Effect of single amino acid substitutions on vasoinhibin isoforms generation.

Vi-isoform	Enzyme/Subst.	Amino acid position/P-position/Amino acid/number of observed cleavages (MEROPS)								8P-score	8P-change
16.8 kDa		172	173	174	175	176	177	178	179		
		P4	P3	P2	P1	P1’	P2’	P3’	P4’		
		Asn	Glu	Ile	Tyr	Pro	Val	Trp	Ser		
	CatD	29	104	70	37	3	98	3	65	409	
	MMP-8	3	3	1	4	0	7	1	6	25	
	MMP-13	2	1	2	1	0	12	0	4	22	
	SER > LEU	P4	P3	P2	P1	P1’	P2’	P3’	P4’		
	rs142454996	Asn	Glu	Ile	Tyr	Pro	Val	Trp	Leu		
	CatD	29	104	70	37	3	98	3	63	407	−2
	MMP-8	3	3	1	4	0	7	1	13	32	+7
	MMP-13	2	1	2	1	0	12	0	12	30	+8
	TRP > CYS	P4	P3	P2	P1	P1’	P2’	P3’	P4’		
	rs373557237	Asn	Glu	Ile	Tyr	Pro	Val	Cys	Ser		
	CatD	29	104	70	37	3	98	11	65	417	+8
	MMP-8	3	3	1	4	0	7	2	6	26	+1
	MMP-13	2	1	2	1	0	12	0	4	22	

17.2 kDa		175	176	177	178	179	180	181	182		
		P4	P3	P2	P1	P1’	P2’	P3’	P4’		
		Tyr	Pro	Val	Trp	Ser	Gly	Leu	Pro		
	CatD	33	30	137	29	41	56	112	55	493	
	TRP > CYS	P4	P3	P2	P1	P1’	P2’	P3’	P4’		
	rs373557237	Tyr	Pro	Val	Cys	Ser	Gly	Leu	Pro		
	CatD	33	30	137	8	41	56	112	55	472	−21
											
	SER > LEU	P4	P3	P2	P1	P1’	P2’	P3’	P4’		
	rs142454996	Tyr	Pro	Val	Trp	Leu	Gly	Leu	Pro		
	CatD	33	30	137	29	110	56	112	55	562	+69

17.7 kDa		180	181	182	183	184	185	186	187		
		P4	P3	P2	P1	P1’	P2’	P3’	P4’		
		Gly	Leu	Pro	Ser	Leu	Gln	Met	Ala		
	MMP-8	36	7	9	6	37	12	0	13	120	
	MMP-13	49	9	20	11	49	13	1	7	159	
	SER > ALA	P4	P3	P2	P1	P1’	P2’	P3’	P4’		
	rs138984819	Gly	Leu	Pro	Ala	Leu	Gln	Met	Ala		
	MMP-8	36	7	9	8	37	12	0	13	122	+2
	MMP-13	49	9	20	13	49	13	1	7	161	+2

15 kDa		157	158	159	160	161	162	163	164		
		P4	P3	P2	P1	P1’	P2’	P3’	P4’		
		Gly	Met	Glu	Leu	Ile	Val	Ser	Gln		
	CatD	42	26	94	416	119	131	55	52	935	
	MET > VAL	P4	P3	P2	P1	P1’	P2’	P3’	P4’		
	rs749978269	Gly	Val	Glu	Leu	Ile	Val	Ser	Gln		
	CatD	42	94	94	416	119	131	55	52	1,003	+68

*Six reported point mutations leading to amino acid substitutions in vasoinhibin-generating cleavage sites were retrieved from ENSEMBL (25). An 8P-score indicating the proteolysis efficiency of vasoinhibin-generating enzymes was calculated for the ancestral sequence and the sequence containing point mutations. The amino acid substitutions in the cleavage sites either facilitate or inhibit the generation of the respective isoform which is indicated by an increase or decrease of the 8P-score. The molecular weight of the vasoinhibin isoforms is shaded in gray, as are the point mutations in the cleavage sites*.

**Table 2:** Effect of single amino acid substitutions observed in cancer on vasoinhibin isoforms generation.

Vi-isoform	Enzyme/Subst.	Amino acid position/P-position/Amino acid/number of observed cleavages (MEROPS)								8P-score	8P-change
16.8 kDa		172	173	174	175	176	177	178	179		
		P4	P3	P2	P1	P1’	P2’	P3’	P4’		
		Asn	Glu	Ile	Tyr	Pro	Val	Trp	Ser		
	CatD	29	104	70	37	3	98	3	65	409	
	MMP-8	3	3	1	4	0	7	1	6	25	
	MMP-13	2	1	2	1	0	12	0	4	22	
		Hepatocellular carcinoma (29)									

	PRO > ALA	P4	P3	P2	P1	P1’	P2’	P3’	P4’		
	COSM1727363	Asn	Glu	Ile	Tyr	Ala	Val	Trp	Ser		
	CatD	29	104	70	37	83	98	3	65	489	+80
	MMP-8	3	3	1	4	8	7	1	6	33	+8
	MMP-13	2	1	2	1	9	12	0	4	31	+9
		Lung adenocarcinoma (30)									

	PRO > HIS	P4	P3	P2	P1	P1’	P2’	P3’	P4’		
	COSM336863	Asn	Glu	Ile	Tyr	His	Val	Trp	Ser		
	CatD	29	104	70	37	0	98	3	65	406	-3
	MMP-8	3	3	1	4	2	7	1	6	27	+2
	MMP-13	2	1	2	1	2	12	0	4	24	+2

17.2 kDa		175	176	177	178	179	180	181	182		
		P4	P3	P2	P1	P1’	P2’	P3’	P4’		
		Tyr	Pro	Val	Trp	Ser	Gly	Leu	Pro		
	CatD	33	30	137	29	41	56	112	55	493	
		Gastric cancer (31)									

	GLY > GLU	P4	P3	P2	P1	P1’	P2’	P3’	P4’		
	COSM4751148	Tyr	Pro	Val	Trp	Ser	Glu	Leu	Pro		
	CatD	33	30	137	29	41	81	112	55	518	+25

17.7 kDa		180	181	182	183	184	185	186	187		
		P4	P3	P2	P1	P1’	P2’	P3’	P4’		
		Gly	Leu	Pro	Ser	Leu	Gln	Met	Ala		
	MMP-8	36	7	9	6	37	12	0	13	120	
	MMP-13	49	9	20	11	49	13	1	7	159	
		Small-cell lung cancer (32, 33)									

	ALA > THR	P4	P3	P2	P1	P1’	P2’	P3’	P4’		
	COSM314455	Gly	Leu	Pro	Ser	Leu	Gln	Met	Thr		
	MMP-8	36	7	9	6	37	12	0	8	115	−5
	MMP-13	49	9	20	11	49	13	1	7	159	–

*Four reported point mutations in cancer tissues (hepatocellular carcinoma, lung adenocarcinoma, gastric cancer, and small-cell lung cancer) leading to amino acid substitutions in vasoinhibin-generating cleavage sites were retrieved from the COSMIC database (27). An 8P-score indicating the proteolysis efficiency of vasoinhibin-generating enzymes was calculated for the ancestral sequence and the sequence containing point mutations. The amino acid substitutions in the cleavage sites either facilitate or inhibit the generation of the respective isoform which is indicated by an increase or decrease of the 8P-score. The molecular weight of the vasoinhibin isoforms is shaded in gray, as are the point mutations in the cleavage sites*.

**Figure 2:**
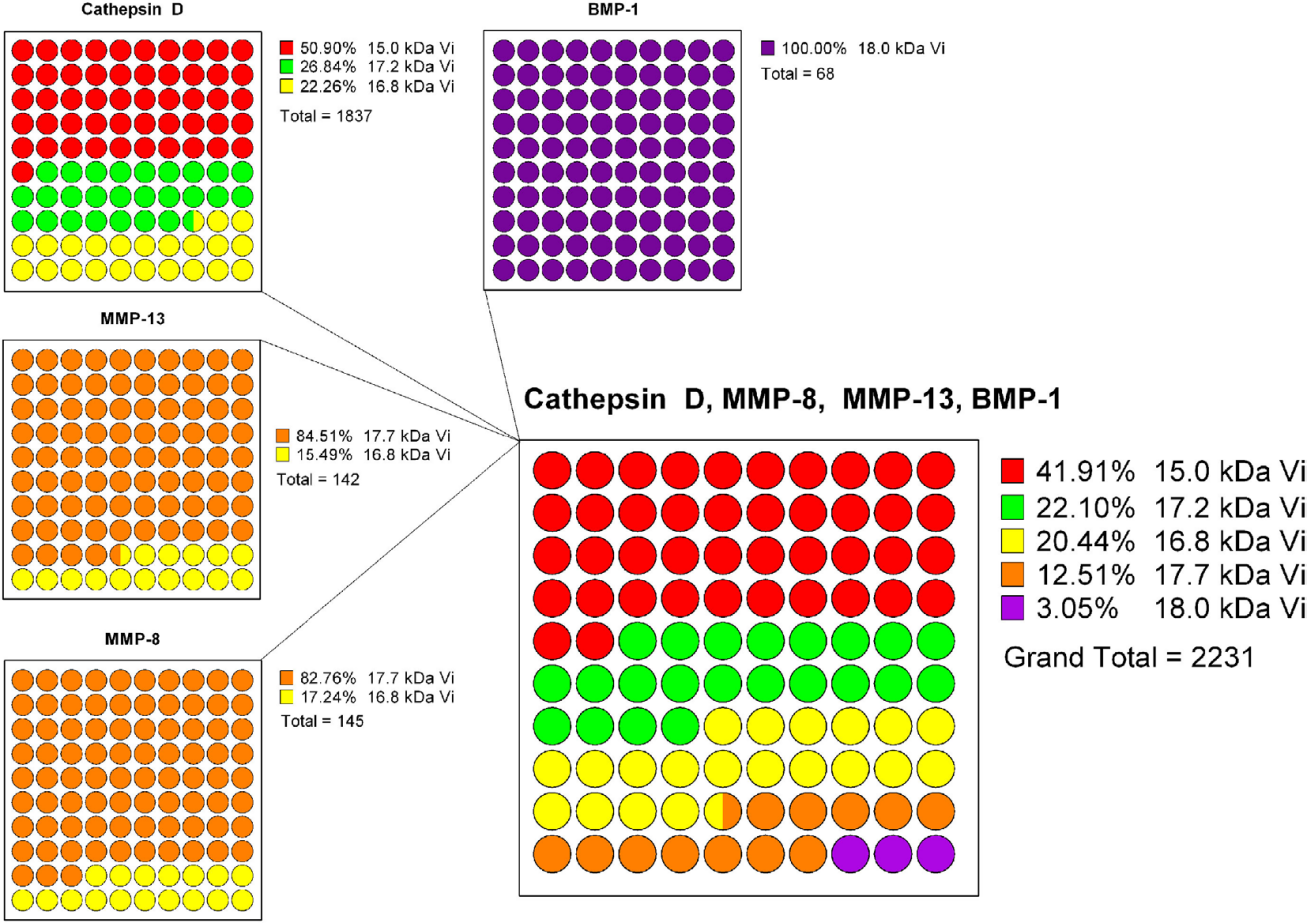
To estimate the composition of vasoinhibin isoforms, a projection based on the known prolactin-cleavage sites of vasoinhibin-generating enzymes (Cathepsin D, MMP-8/13, and BMP-1) and their calculated cleavage efficiency (8P-score) was performed. Cathepsin D generates the 15, the 17.2, and the 16.8 kDa vasoinhibin isoform, with the highest cleavage efficiency at the site generating the 15 kDa isoform. The 8P-scores of each Cathepsin D cleavage site were added and represented in parts of a whole graph. The same analysis was performed for MMP-13, MMP-8, and BMP-1. Finally, the percent representation of the cleavage efficiency of all enzymes and vasoinhibin isoforms was combined. It appears that, on the basis of the cleavage efficiency of vasoinhibin-generating enzymes, the 15 kDa vasoinhibin isoform would be most abundant (41.91%), followed by the 17.2 kDa (22.10%), the 16.8 kDa (20.44%), the 17.7 kDa (12.51%), and the 18 kDa (3.05%) isoform.

A projection of the relative composition of vasoinhibin isoforms was performed, showing the relative proteolysis efficiency of four PRL-cleaving enzymes and the corresponding vasoinhibin isoform generated, respectively (Figure 2). From this projection, it is most likely that the most abundant isoform is the 15 kDa vasoinhibin, followed by the 17.2 kDa, and the 16.8 kDa isoform, all of which are generated by cathepsin D, and MMP-8 and MMP-13 in case of the 16.8 kDa isoform. The 17.7 and 18 kDa vasoinhibin isoforms demonstrate much lower scores and are, thus, likely to be less abundant (Figure 1). Of note, the general order of composition 15 > 17.2 > 16.8 > 17.7 > 18 kDa isoform is maintained in all scenarios considering all mutations tested, although with slight variation.

## Discussion

The present analysis demonstrates that besides substrate (PRL) availability and enzyme activity, point mutations in the human PRL gene leading to amino acid substitutions in cleavage sites may determine vasoinhibin levels. The amino acid substitutions appear to influence enzymatic cleavage of PRL, as some amino acids facilitate the cleavage, whereas others reduce the cleavage efficiency. This is a relevant finding, as factors and controlling mechanisms governing the generation of vasoinhibins are incompletely understood.

### Methodological Considerations

The present analysis is based on the enzyme-substrate specificity data from the MEROPS database of proteolytic enzymes, in which data from experimental observations are listed. Hence, the projection of the 8P-score, as performed in the present analysis, is based on experimental evidence. However, it remains a projection and is as such theoretical, especially since the 8P-score is a compound score summarizing the effects observed on 8 amino acid positions, which may not necessarily be representative and complex interactions arising from the complete cleavage site sequence, which are independent from the calculated score, may be missed. Also, since the 8P-score is based on the linear sequence of amino acids, effects arising from the three-dimensional structure of the protein in solution, such as effects of the organizational degree of the amino acids at the cleavage site or their various degrees of solvent exposure, are not being considered. Hence, these projections should be tested under experimental conditions evaluating the proteolysis of PRL with and without point mutations at cleavage site regions. Piwnica et al. (12) reported some substitutions and their effects on vasoinhibin generation. They showed that substitution of Leucine with Lysine at position 160 abolishes the cathepsin D cleavage generating the 15 kDa vasoinhibin isoform. This experimental observation corresponds with the 8P-score used in this study, as the score demonstrates a decline from 935 in the ancestral sequence to 525 (−410) in the sequence with p.L160K. Another observation by Piwnica et al. was that the substitution of proline by leucine at position 176 dramatically increases cleavage efficiency. Again, this is confirmed by corresponding changes in the 8P-score, which increases by +107 (16.8 kDa vasoinhibin, cathepsin D), +37 (MMP-8) and +49 (MMP-13), and +69 (17.2 kDa vasoinhibin, cathepsin D), respectively. Finally, the observation by Piwnica et al. of an increased cleavage by substitution of Proline at position 176 by alanine corresponds with respective changes in the 8P-score (16.8 kDa vasoinhibin: +80 for cathepsin D, +8 for MMP-8, +9 for MMP-13; 17.2 kDa vasoinhibin: +5 for cathepsin D).

There are additional vasoinhibin isoforms smaller than 15 kDa that were not considered in the present study since no reliable information about their precise PRL-cleavage sites, either by sequencing or by mass spectrometry, could be retrieved. Also, other MMP (MMP-3, 1, 2, and 9) known to generate vasoinhibins (11) were not considered and the consequences of the mutations in PRL sequence might be different from those projected for MMP-8 and 13. The latter were selected since they cleave PRL with the highest relative rate.

### PRL Point Mutations May Influence Vasoinhibin-Related Diseases

The present analysis proposes that point mutations in the PRL gene, which result in single amino acid substitutions in the PRL sequence, impact the levels and isoform composition of vasoinhibins by altering the efficacy of PRL-cleaving enzymes. These point mutations may, therefore, constitute risk or protective factors affecting diseases associated with a dysregulation of the PRL/vasoinhibin axis in terms of deficient or excessive vasoinhibin generation. One such disease is PPCM, defined by the Heart Failure Association of the European Society of Cardiology Working Group on Peripartum Cardiomyopathy (PPCM) as: “PPCM is an idiopathic cardiomyopathy presenting with heart failure secondary to left ventricular systolic dysfunction toward the end of pregnancy or in the month following delivery, where no other cause of heart failure is found. It is a diagnosis of exclusion. The left ventricle may not be dilated but the ejection fraction (EF) is nearly always reduced below 45%” (34). PPCM is a rare disease, which occurred with a frequency of 1 case/3189 live births and an estimated mortality of 1.36–2.05% (confidence interval 0.29–10.8%) from 1990 to 2002 in the United States (35). Age, race, multiple gestations, and geographical region are associated factors recently reviewed and not discussed here (36–39). As mentioned in the above definition of PPCM, the disease is idiopathic, meaning that no specific causal pathological mechanism could be identified. However, mechanistic research revealed that the 16 kDa vasoinhibin isoform, also referred to as 16 kDa PRL, could be causally related to the development of PPCM (10). PRL, colloquially referred to as the “nursing hormone,” physiologically rises during pregnancy in preparation for lactation and remains elevated in the postpartum period (40). Because of the known, potentially detrimental effects of vasoinhibins on blood vessels (41–43), it was suggested that a higher than normal cleavage of PRL, resulting in an excessive generation of the 16 kDa vasoinhibin isoform, impairs myocardial microvascularization and is a causal trigger of PPCM (10). Consistent with this notion, a higher activity of the vasoinhibin-generating enzyme cathepsin D was demonstrated in the plasma of patients with PPCM (10), and a new therapy for PPCM using the dopamine D2 receptor agonists, cabergoline, and bromocriptine is currently being evaluated in a multicenter clinical trial (NCT00998556) (17). The concept underlying this putative therapy is the inhibition of the generation of vasoinhibins by substrate depletion, i.e., the inhibition of pituitary PRL secretion by activation of dopamine D2 receptors in lactotrophs. However, even if the vasoinhibin-related concept of PPCM etiopathology is substantiated, unanswered questions remain. Despite consideration of known associated factors, the risk of PPCM cannot be reliably predicted and the clinical course is variable, ranging from relatively mild cases to more severe, prolonged cases and also lethal outcomes. It will remain an open question why patients with similar risk factors—high PRL levels and high PRL-cleaving enzyme activity—could experience either no, mild, or severe PPCM. A missense PRL variant affecting the sequence at which proteases act to generate vasoinhibins, could increase cleavage to vasoinhibins, and may constitute a “second hit” in the pathogenesis of PPCM; whereas a variant that results in decreased cleavage may constitute a protective factor, as, despite high activity of cleaving enzymes, the levels of vasoinhibins generated would be lower and less detrimental effects would be expected.

A similar concept can be applied to diabetic retinopathy, in which vasoinhibins play a protective role in terms of their inhibitory effect on retinal neovascularizations, a hallmark of proliferative diabetic retinopathy. Furthermore, vasoinhibins participate in maintaining the normal retinal vasculature (44) and corneal avascularity (45). Raising circulating PRL levels leads to vasoinhibin accumulation in the retina (9), and the elevation of intraocular vasoinhibins prevents and reverses diabetes-induced excessive retinal vasopermeability in rats (46, 47) and angiogenesis in a mouse model of retinopathy of prematurity (48). Also, immunosequestering ocular vasoinhibins stimulates adult rat retinal angiogenesis (44) and reduces the apoptosis of the ocular hyaloid vasculature in neonatal rats (49). As in the etiopathology of PPCM, PRL point mutations affecting the amino acid sequence at vasoinhibin-generating cleavage sites, may, by modifying their levels and composition, enhance or diminish vasoinhibin effects. In consequence, these mutations may account for inter-individual differences in scenarios in which PRL levels as well as enzyme activities are similar, but observed vasoinhibin-related effects vary.

Four mutations in cancer tissues likely affecting the generation of vasoinhibins were found using the COSMIC database. Three of these mutations facilitated PRL-cleavage, whereas a fourth demonstrated a minor negative effect (Table 2). This is of relevance as vasoinhibins (the 16 kDa vasoinhibin isoform) reduced angiogenesis in a breast cancer model (50) and adenovirus-mediated vasoinhibin expression in prostate cancer cells lowered their ability to form tumors in a xenograft animal model (51). Another study reported that the 16 kDa vasoinhibin isoform reduced the growth and microvascular density of tumors derived from implanted human colon cancer cells in mice (52). Furthermore, adenovirus-mediated gene transfer of the 16 kDa vasoinhibin inhibited tumor growth in a subcutaneous mouse melanoma model and reduced the size and number of lung metastases (53). Considering these studies, the generation of vasoinhibins in cancer tissues may be a factor debilitating tumor fitness because two classical hallmarks of cancer (54)—the induction of angiogenesis and the activation of invasion and metastasis—are compromised. On the other hand, vasoinhibins can act as potent proinflammatory cytokines stimulating iNOS expression and NO production by pulmonary fibroblasts (5, 55) and these effects may contribute to inflammation-mediated restrain of tumor growth.

### Composition of Vasoinhibin Isoforms

Studies reported the detection of one, two, or several vasoinhibin isoforms in biological fluids such as plasma and urine from patients, but also in tissue lysates (15, 18, 20, 56). This alone indicates that the composition of vasoinhibins varies during physiological states and disease conditions. Such variation is to be expected because the type of PRL cleavage depends on the cellular and extracellular location and activity of vasoinhibin-generating enzymes, and on additional microenvironmental factors affecting enzyme kinetics, such as pH and temperature (57). We did not consider these additional factors, which is why the projected vasoinhibin composition of 15 > 17.2 > 16.8 > 17.7 > 18 kDa isoform is only a rough approximation of what can be expected to occur *in vivo*. However, the projection is useful as it points to the need to consider more than one vasoinhibin isoform when detecting vasoinhibins in biological fluids. Furthermore, the projected composition question investigations in which isoforms were assigned the molecular mass of 16.8 kDa (3, 10), because the 15 and 17.2 kDa vasoinhibin isoforms are projected to be more abundant. Moreover, if only one cathepsin D cleaved isoform is detected, mechanisms must be present suppressing the generation of the other cathepsin D-generated isoforms (58). The problem extends to studies of vasoinhibin-related diseases and points out that differentiation between vasoinhibin isoforms and complete characterization of their generation process should be a focus in future investigations (22, 23). This is a prerequisite to resolve uncertainties about which isoforms should be targeted when manufacturing anti-vasoinhibin monoclonal antibodies for the development of quantitative immunoassays and for further characterization of vasoinhibin physiology and related diseases. The general order of vasoinhibin composition 15 > 17.2 > 16.8 > 17.7 > 18 kDa isoform did not change in consequence of a mutation. Hence, the mutations appear more relevant for the levels of single vasoinhibin isoforms and not for the general order of vasoinhibin composition. However, the large change (+69 points) seen in the 8P-score in case of the 17.2 kDa isoform, when SER is substituted by LEU in position 1 (Table 1), for example, demonstrates that other mutations not yet detected may indeed change the general order of vasoinhibin composition. Whether such change would be relevant or not cannot be ascertained momentarily, as comparative analyses of mixtures of vasoinhibin isoforms have not yet been performed.

## Conclusion

Our work proposes that—besides substrate availability and enzyme activity—point mutations in the PRL gene may impact vasoinhibin generation and vasoinhibin-related disease outcomes. This hypothesis is testable as point mutations affecting cleavage site regions can be evaluated in patients with vasoinhibin-related diseases and the proteolytic cleavage of PRL carrying these mutations can be performed in *in vitro* assays. The study of only one vasoinhibin isoform when investigating vasoinhibin levels in patients appears insufficient. This underscores the need to heighten the focus on the neglected issue of addressing the various vasoinhibin isoforms and to differentiate between them. This will facilitate the ongoing process of understanding the functional role of vasoinhibins under health and disease.

## Author Contributions

JT designed research and wrote the manuscript. AL, CF, and JT performed data analysis. GE, CC, and TB edited and revised the manuscript. All authors approved the final version of the manuscript.

## Conflict of Interest Statement

The authors declare that the research was conducted in the absence of any commercial or financial relationships that could be construed as a potential conflict of interest.

## Footnotes

^1^http://www.ensembl.org.

^2^http://cancer.sanger.ac.uk/cosmic.

^3^http://merops.sanger.ac.uk.
